# PLEKHA7 Is an Adherens Junction Protein with a Tissue Distribution and Subcellular Localization Distinct from ZO-1 and E-Cadherin

**DOI:** 10.1371/journal.pone.0012207

**Published:** 2010-08-20

**Authors:** Pamela Pulimeno, Christoph Bauer, Jeffrey Stutz, Sandra Citi

**Affiliations:** 1 Department of Molecular Biology, University of Geneva, Geneva, Switzerland; 2 National Centre of Competence in Research (NCCR) Frontiers in Genetics, University of Geneva, Geneva, Switzerland; CNRS UMR6543, Université de Nice, France

## Abstract

The pleckstrin-homology-domain-containing protein PLEKHA7 was recently identified as a protein linking the E-cadherin-p120 ctn complex to the microtubule cytoskeleton. Here we characterize the expression, tissue distribution and subcellular localization of PLEKHA7 by immunoblotting, immunofluorescence microscopy, immunoelectron microscopy, and northern blotting in mammalian tissues. Anti-PLEKHA7 antibodies label the junctional regions of cultured kidney epithelial cells by immunofluorescence microscopy, and major polypeptides of M_r_ ∼135 kDa and ∼145 kDa by immunoblotting of lysates of cells and tissues. Two PLEKHA7 transcripts (∼5.5 kb and ∼6.5 kb) are detected in epithelial tissues. PLEKHA7 is detected at epithelial junctions in sections of kidney, liver, pancreas, intestine, retina, and cornea, and its tissue distribution and subcellular localization are distinct from ZO-1. For example, PLEKHA7 is not detected within kidney glomeruli. Similarly to E-cadherin, p120 ctn, β-catenin and α-catenin, PLEKHA7 is concentrated in the apical junctional belt, but unlike these adherens junction markers, and similarly to afadin, PLEKHA7 is not localized along the lateral region of polarized epithelial cells. Immunoelectron microscopy definitively establishes that PLEKHA7 is localized at the adherens junctions in colonic epithelial cells, at a mean distance of 28 nm from the plasma membrane. In summary, we show that PLEKHA7 is a cytoplasmic component of the epithelial adherens junction belt, with a subcellular localization and tissue distribution that is distinct from that of ZO-1 and most AJ proteins, and we provide the first description of its distribution and localization in several tissues.

## Introduction

Epithelial cells are characterized by an apical junctional complex, comprising tight junctions (TJ), adherens junctions (AJ) and desmosomes [1]. TJ and AJ are of critical importance in the development and physiology of vertebrate epithelial tissues. TJ control the barrier function of epithelia and maintain cell polarity, and AJ regulate cell-cell adhesion and morphogenesis [2], [3]. The molecular architecture of TJ and AJ has been largely clarified in recent years [4], [5], [6], [7], [8]: junctions comprise transmembrane proteins that are linked to cytoskeletal filaments through a cytoplasmic plaque, that contains scaffolding, adaptor and signaling proteins.

The cytoskeleton plays a fundamental role in the regulation of the assembly and function of TJ and AJ. Actomyosin filaments modulate the TJ barrier and orchestrate the signaling and adhesive functions of AJ, through the interaction with several actin-binding junctional proteins, including Zonula-Occludens-1 (ZO-1), α-catenin, and afadin [9], [10], [11], [12]. Microtubules are implicated in membrane remodeling and polarized vesicle traffic, influencing the barrier and adhesive functions of TJ and AJ [13], [14], [15], [16]. Little is known about the molecules that interact with microtubules at epithelial junctions [16], [17], [18]. Recently, it was shown that E-cadherin, the major transmembrane protein of AJ, associates with microtubules through a protein complex comprising p120 ctn, and the newly identified proteins PLEKHA7 and nezha [19]. Although it was shown that PLEKHA7 partially co-localizes with E-cadherin in cultured intestinal cells [19], nothing is known about its pattern of expression and its subcellular localization in epithelial tissues. Furthermore, the localization of PLEKHA7 at the ultrastructural level has not been determined in any tissue.

To address these questions, we used northern blot analysis, immunoblotting, immunofluorescence and immunoelectron microscopy with specific antibodies that we generated and characterized. The results show that PLEKHA7 is localized at epithelial AJ, has a localization distinct from ZO-1 and most markers of the AJ plaque, and exists in multiple isoforms.

## Results

### Specific antibodies against PLEKHA7 label the junctional regions of cultured renal epithelial cells and detect M_r_ ∼145 and ∼135 kDa polypeptides in cell and tissue lysates

**Figure 1:**
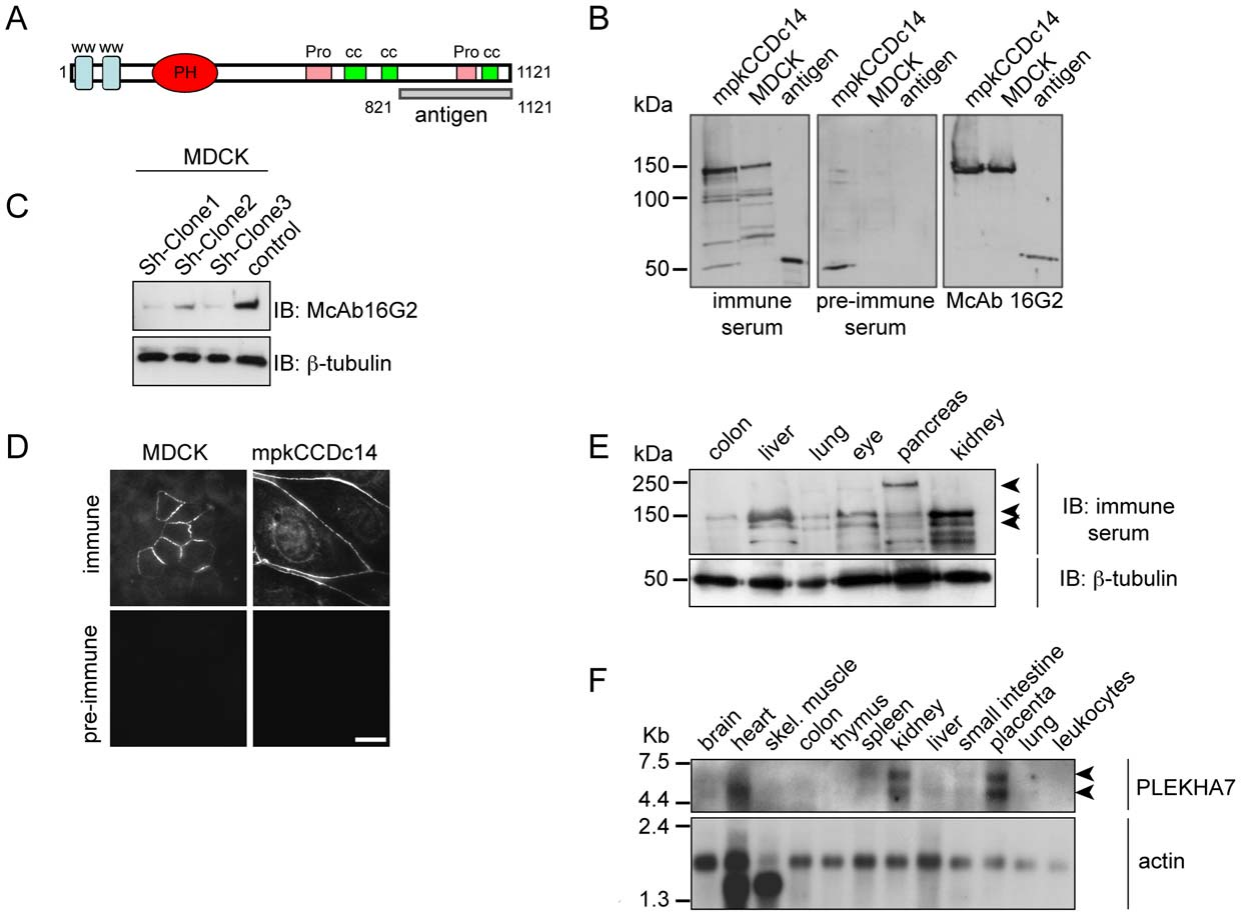
Characterization of anti-PLEKHA7 antibodies, and expression of PLEKHA7 in cells and tissues. **A.** Schematic diagram of the domain organization of PLEKHA7, showing WW, pleckstrin-homology (PH), proline-rich (Pro), and coiled-coil (cc) domains. The C-terminal region comprising the sequences of PLEKHA7 used to generate the GST-fusion protein is indicated (antigen, residues 821–1121). **B.** Immunoblotting analysis of lysates of mouse (mpkCCDc14) and dog (MDCK) renal epithelial cells, and PLEKHA7 antigen (see (A)) using rabbit immune and pre-immune sera, and mouse monoclonal antibody 16G2. A polypeptide of M_r_ ∼145 kDa and the antigen (∼ 50 kDa) are specifically labeled by both antibodies. In addition, the immune serum labels additional polypeptides of smaller size, which may in part derive from proteolytic degradation of full-length PLEKHA7, and in part from non-specific cross-reaction. Apparent sizes of molecular size markers are indicated (kDa). **C.** Immunoblotting analysis of lysates of three clonal lines of MDCK cells depleted of PLEKHA7 by shRNA-mediated silencing, using the monoclonal antibody 16G2, or anti-β-tubulin antibodies (to normalize protein loadings). **D.** Immunofluorescence microscopy analysis of exogenous human PLEKHA7 expressed in canine MDCK cells (left) and endogenous PLEKHA7 in mouse mpkCCDc14 cells (right), using either immune or pre-immune rabbit serum (Bar = 15 µm). Note that by immunofluorescence microscopy the rabbit immune serum does not recognize canine PLEKHA7, but only the exogenously expressed human protein. **E.** Immunoblotting analysis of PLEKHA7 in lysates of epithelial tissues, using either polyclonal anti-PLEKHA7 immune serum, or anti-β-tubulin antibodies. Arrowheads on the right indicate major polypeptides labeled by the polyclonal antiserum, with apparent sizes of M_r_ ∼240 kDa, M_r_ ∼145 kDa, and M_r_ ∼135 kDa. **F.** Northern blot analysis of a human multiple tissue array of total RNA, hybridized using specific PLEKHA7 (top panel) and actin (bottom panel) DIG-labelled probes. Arrowheads on the right indicate that PLEKHA7 mRNA is detected as two bands of ∼5.5 kb and ∼6.5 kb.

PLEKHA7 contains two WW domains and one pleckstrin homology (PH) domain in the N-terminal half, and coiled-coil (cc) and proline-rich domains in the C-terminal half of the molecule (Fig. 1A) [19]. We generated polyclonal and monoclonal antibodies against a C-terminal fragment of human PLEKHA7, comprising one proline-rich and one coiled-coil domain (“antigen”, Fig. 1A), fused to glutathione-S-transferase (GST). By immunoblotting, the antibodies labelled a major polypeptide, with an apparent size of M_r_ ∼145 kDa in lysates of epithelial cells from mouse (mpkCCDc14) and dog (MDCK) kidney, as well as the antigen (∼50 kDa) (Fig. 1B). The signal of the M_r_ ∼145 kDa polypeptide was decreased in MDCK cells depleted of PLEKHA7 by shRNA-mediated interference (Fig. 1C). The epitope recognized by the monoclonal antibody was mapped within residues 920–1020, as determined by immunoblotting of bacterially expressed fragments of the PLEKHA7 antigen (data not shown). By immunofluorescence microscopy, the polyclonal antibodies specifically labelled the region of cell-cell contact in MDCK cells expressing exogenous human PLEKHA7, and the endogenous protein in mpkCCDc14 cells (Fig. 1D). These results are in agreement with previous work, reporting the junctional localization and molecular size of PLEKHA7 in cultured human intestinal cells [19].

Immunoblot analysis of lysates of different epithelial tissues (colon, liver, lung, eye, pancreas, kidney) and heart tissue showed major polypeptides of M_r_ ∼145 kDa and ∼135 kDa (Fig. 1E, and data not shown). A polypeptide with apparent size of M_r_ ∼240 kDa was clearly labelled in lysates of pancreas, by both polyclonal antiserum and monoclonal antibody, and was also detectable in lysates of lung, eye and liver (Fig. 1E, and data not shown). Such large polypeptide was not detected in lysates of cultured cell lines, and its detection is probably due to the presence of cross-reacting epitopes in an unrelated protein (see Discussion).

To establish the size and pattern of tissue expression of PLEKHA7 transcripts, we performed a northern blot analysis of RNA from several tissues. PLEKHA7 mRNA was detected as two major transcripts, with apparent sizes of ∼5.5 kb and ∼6.5 kb, in brain, kidney, liver, small intestine, placenta and lung, and as one major transcript of 5.5 kb in the heart and other tissues (Fig. 1F). Signal intensity was high in the placenta, kidney and heart samples, and low in brain, colon, liver, skeletal muscle, spleen, small intestine and lung samples, whereas no signal was detected in thymus and leukocytes samples (Fig. 1F). Taken together, these results show that the antibodies that we generated are specific for PLEKHA7, and indicate that PLEKHA7 exists in multiple isoforms.

### The tissue distribution and junctional localization of PLEKHA7 in epithelial tissues is distinct from ZO-1

**Figure 2:**
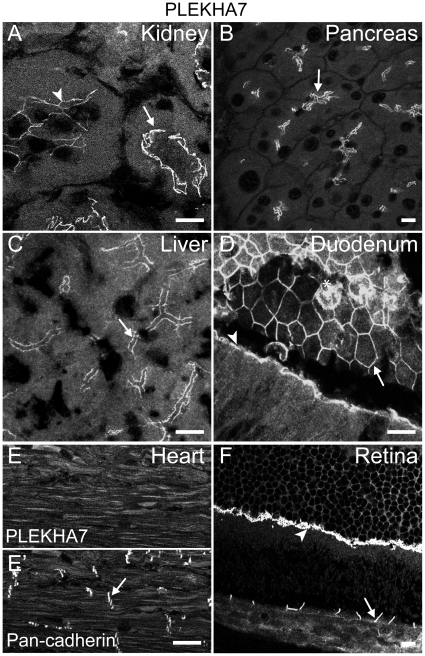
PLEKHA7 is localized at cell-cell junctions in epithelial tissues. Immunofluorescent analysis of PLEKHA7 distribution in kidney (A), pancreas (B), liver (C), duodenum (D), heart (E) and retina (F), using polyclonal immune serum, and pan-cadherin distribution in heart (E′). Arrows indicate junctions between epithelial cells, except in E′, where the arrow indicates intercalated disks. The arrowhead in A indicates weaker junctional labeling in a subset of cortical tubules. The arrowhead in D indicates apical staining in longitudinally sectioned columnar epithelial cells. The asterisk in D indicates non-specific labeling of mucus in goblet cells. The arrowhead in F indicates staining in the outer limiting membrane of the retina. Bar = 10 µm.

To determine the localization of PLEKHA7, frozen sections of epithelial tissues and heart sections were labeled with anti-PLEKHA7 antibodies. Immunofluorescence microscopy analysis showed labeling of junctions in the kidney, pancreas, liver, duodenum, ileum, retina, and lung (Fig. 2, and data not shown). In the kidney cortex, epithelial cells of convoluted tubules were labeled, with a subset of tubules displaying slightly weaker staining (arrowhead in Fig. 2A) than others (arrow in Fig. 2A). In the pancreas, epithelial cells of the acini and ductules showed positive staining (arrow in Fig. 2B), whereas no labeling of the islets of Langerhans was detected (data not shown). Similarly, in sections of liver, labeling was detected along the junctions between epithelial cells lining bile canaliculi (arrow in Fig. 2C). In the duodenum (Fig. 2D), colon and ileum (see below and data not shown), labeling appeared as a thin apical bar in longitudinal sections (arrowhead in Fig. 2D), and as a net-like meshwork in tangential sections (arrow in Fig. 2D, and data not shown) of enterocytes. In heart sections, anti-PLEKHA7 antibodies gave diffuse staining of cardiac myocites, without any accumulation at identifiable structures (Fig. 2E), whereas in the same sections anti-cadherin antibodies prominently labeled the intercalated disks between cardiac myocites (Fig. 2E′). In the retina, labeling was detected in the outer limiting membrane (OLM) (arrowhead in Fig. 2F), which has been previously shown to contain both AJ and TJ proteins [20], and along the junctions between retinal pigmented epithelial cells (arrow in Fig. 2F). No PLEKHA7 staining was detected in sections of spleen (data not shown).

**Figure 3:**
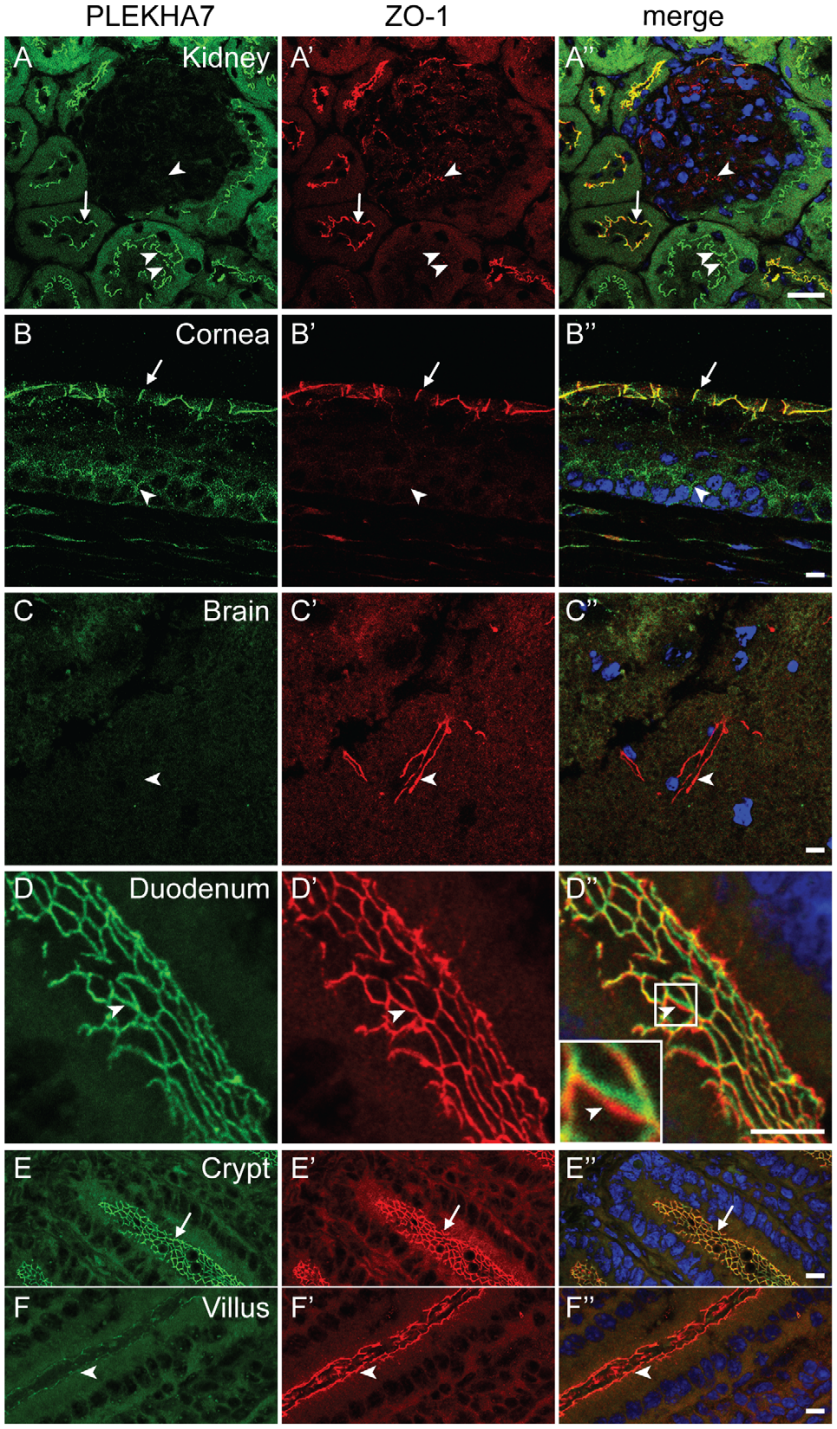
The localization and distribution of PLEKHA7 and ZO-1 are distinct. Double immunofluorescence labeling of PLEKHA7 and ZO-1 in mouse kidney cortex (A-A”), cornea (B-B”), brain (C-C”), and duodenum (D-D”, E-E”, F-F”). Arrows indicate junctions where PLEKHA7 and ZO-1 labeling appear co-localized. Arrowheads indicate junctions that show stronger or only labeling for either PLEKHA7 (B-B”) or ZO-1 (A-A”, C-C”, F-F”). Double arrowheads in A-A″ indicate kidney tubules that show weaker ZO-1 labeling. Merge images show nuclei labeled in blue by DAPI. Bar = 10 µm.

Next, we asked whether the distribution and localization of PLEKHA7 was distinguishable from that of ZO-1, a protein that is localized at TJ in polarized epithelial cells, and at AJ in non-epithelial cells [21], [22], [23]. Double immunofluorescence microscopy showed that in the kidney cortex strong ZO-1 labeling was co-localized with PLEKHA7 in epithelial cells lining the tubules (arrow in Fig. 3A-A″), and ZO-1 labeling was weaker in the subset of tubules, that displayed stronger PLEKHA7 labeling (double arrowheads in Fig. 3A-A″). In addition, ZO-1 labeling was detected within glomeruli, in agreement with previous studies, showing that ZO-1 is localized at podocyte filtration slits [24], whereas no PLEKHA7 labeling was detected within glomeruli (arrowhead in Fig. 3A-A″). In corneal tissue, ZO-1 and PLEKHA7 were co-localized linearly along junctions in the outermost layer of the stratified epithelium (arrow in Fig. 3B-B″), whereas only PLEKHA7 was detected in the basal layer of the epithelium, in a punctate, discontinuous pattern (arrowhead in Fig. 3B-B″). In sections of brain tissue, ZO-1 labeling was detected in short linear segments, that may correspond to endothelial junctions of blood vessels, but no corresponding PLEKHA7 labeling was detectable at these sites (arrowhead in Fig. 3C-C″). Tangential sections of the duodenum showed a very similar distribution of ZO-1 and PLEKHA7 (Fig. 3D-D″). However, the labeling for the two proteins could be spatially distinguished given the appropriate section orientation (arrowhead, and magnified inset in Fig. 3D-D″). Finally, the intensity of labeling for both PLEKHA7 and ZO-1 was strong in the basal, crypt areas of intestinal villi (arrows in Fig. 3E-E”). In contrast to the staining in the crypts, the apical region of the villi showed a weaker labeling for PLEKHA7, whereas the labeling for ZO-1 was of similar intensity throughout the villus (arrowheads in Fig. 3F-F”). In summary, the pattern of tissue distribution and subcellular localization of PLEKHA7 is distinct from that of ZO-1.

### In epithelial cells, PLEKHA7 is restricted to the AJ apical belt, and is absent from the lateral membrane

To further examine the junctional localization of PLEKHA7, we examined by double immunofluorescence microscopy tissue sections stained with antibodies against PLEKHA7 and several markers of AJ [6], [25], including the transmembrane protein E-cadherin, and several cytoplasmic plaque AJ proteins: 1) α-catenin, which interacts with the cytoplasmic domain of E-cadherin through β-catenin; 2) β-catenin, which interacts with E-cadherin and α-catenin; 3) p120 ctn, which interacts with the juxtamembrane region of E-cadherin, and with PLEKHA7 [19]; 4) afadin, which interacts with nectin and α-catenin [12].

**Figure 4:**
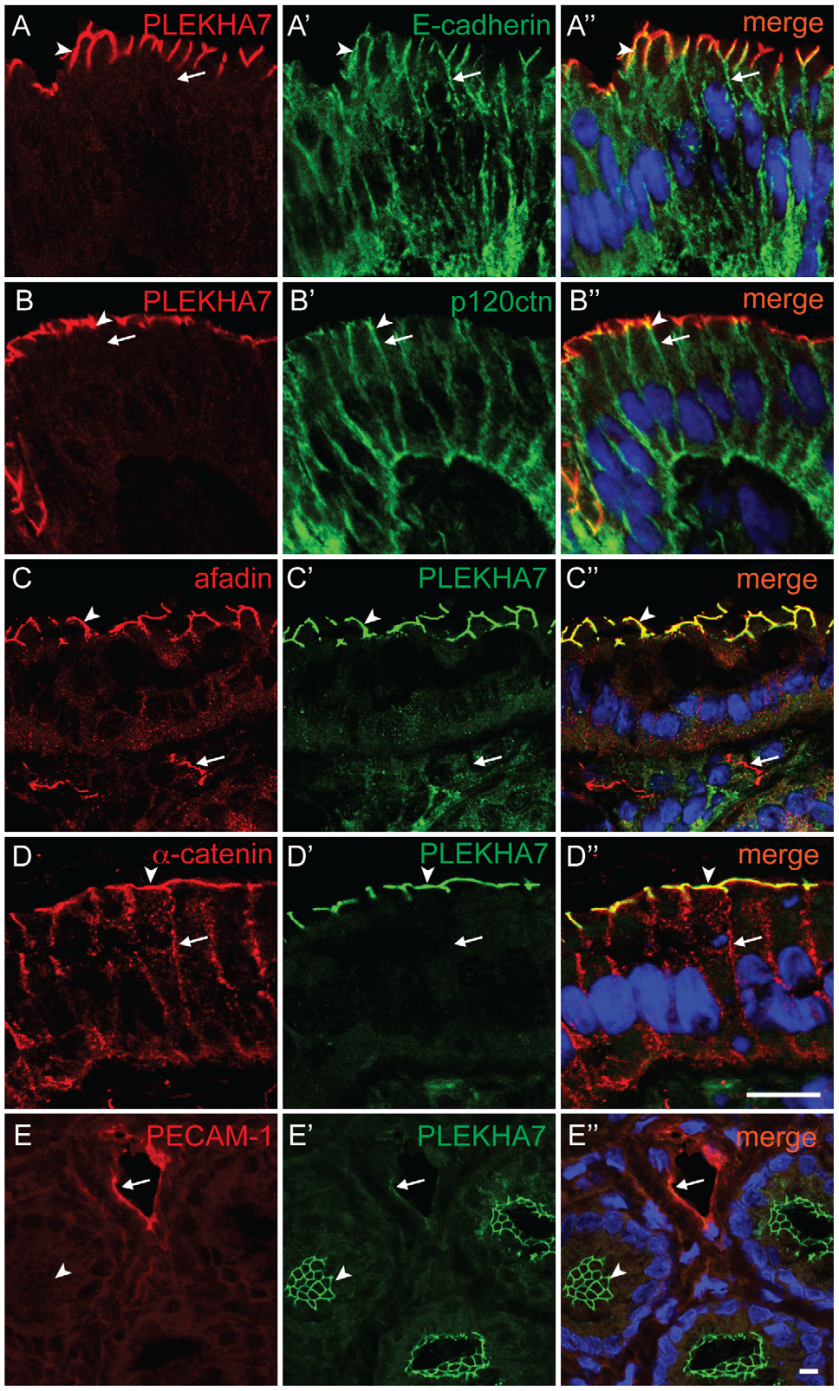
PLEKHA7 colocalizes with AJ proteins at the apical AJ belt, but not along the lateral walls. Double immunofluorescence labeling of sections of intestinal tissue with antibodies against PLEKHA7, together with antibodies against either E-cadherin (A-A”), or p120ctn (B-B″), or afadin (C-C″), or α-catenin (D-D″), or PECAM-1 (E-E″). Arrowheads indicate junctional labeling for PLEKHA7, that is colocalized with the AJ marker (but not in E-E″). Arrows indicate staining of AJ markers along lateral walls (A-A″, B-B″, D-D″). The arrows in C-C″ indicate afadin labeling within the villus stroma (presumably endothelial cells of lymphatic vessels), that is not colocalized with PLEKHA7. Arrows in E-E″ indicate PECAM-1-labeled blood vessels that show very weak PLEKHA7 labeling. Merge images show nuclei labeled in blue by DAPI. Bar = 10 µm.

In sections of colonic villi, PLEKHA7 colocalized at the apical junctions of enterocytes with E-cadherin (arrowhead in Fig. 4A-A″), p120 ctn (arrowhead in Fig. 4B-B″), afadin (arrowhead in Fig. 4C-C″) and α-catenin (arrowhead in Fig. 4D-D″). However, antibodies against E-cadherin (Fig. 4A′), p120ctn (Fig. 4B′) and α-catenin (Fig. 4D), as well as phalloidin and antibodies against β-catenin (data not shown) also labeled the lateral membrane, whereas no labeling for PLEKHA7 was detected in this region (arrows in Fig. 4A-A″, B-B″, D-D″). Conversely, afadin labeling was absent from the lateral membrane, similarly to PLEKHA7, indicating that afadin and PLEKHA7 have an identical localization in these cells (Fig. 4C-C″). However, while afadin was also detected within the villus stroma, presumably along junctions between endothelial cells of lymph vessels, no corresponding labeling for PLEKHA7 was detected (arrows in Fig. 4C-C″). To further examine the localization of PLEKHA7 in blood vessels, we examined its co-localization with the endothelial cell marker PECAM-1. In sections of the intestine near the crypts, the junctions of epithelial cells within the crypts were clearly labeled by anti-PLEKHA7 antibodies (arrowheads in Fig. 4E′-E″), whereas blood vessels showed strong PECAM-1 staining, and very weak PLEKHA7 staining (arrows in Fig. 4E-E″).

**Figure 5:**
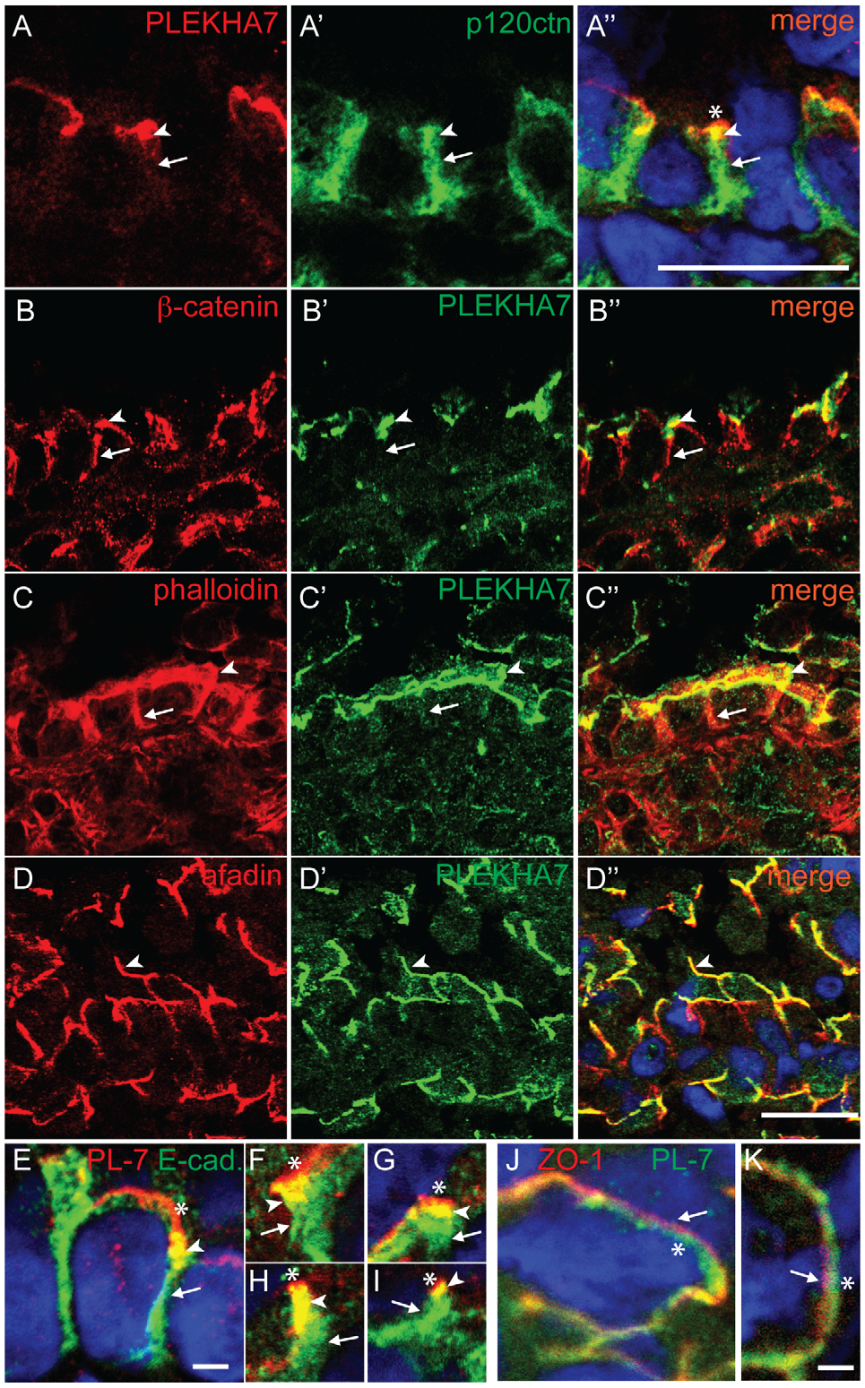
In bronchial epithelial cells PLEKHA7 colocalizes with AJ proteins, but does not localize along lateral walls. Double immunofluorescence labeling of PLEKHA7 with p120ctn (A-A”), β-catenin (B-B″), phalloidin (C-C″), afadin (D-D″), E-cadherin (E-I, merge images only), and ZO-1 (J-K, merge images only) in sections of lung (bronchial cells). Arrowheads indicate colocalization between PLEKHA7 and AJ marker. Arrows indicate staining of AJ markers along lateral walls, that is not colocalized with PLEKHA7, except for panels J-K, where arrows indicate apical staining for ZO-1. Asterisks (E-K) indicate PLEKHA7 labeling apical to the region of colocalization with AJ markers at the AJ belt (E-I), and that is spatially distinct from ZO-1 (J-K). Merge images show nuclei labeled in blue by DAPI. Bar = 10 µm (A-D) and 0.5 µm (E-K).

In bronchial epithelial cells, PLEKHA7 colocalized at apical junctions, but not along the lateral membrane, with p120 ctn (Fig. 5A-A″), β-catenin (Fig. 5B-B″), and E-cadherin (Fig. 5E-I), and showed a localization very similar to afadin (Fig. 5D-D″). Actin filaments were also detected at apical junctions (arrowhead in Fig. 5C-C″) and along lateral membranes, and at this latter location they were not colocalized with PLEKHA7 (arrows in Fig. 5C-C″). In the lung, PLEKHA7 labeling was also detected in the junctions of alveolar pneumocytes (data not shown). Examination of magnified areas of bronchial cells that were double-labelled for E-cadherin and PLEKHA7 showed that the region of colocalization (arrowheads in Fig. 5E, F, G, H, I) was flanked apically by a region containing PLEKHA7 labeling, but devoid of E-cadherin labeling (asterisks in Fig. 5E, F, G, H, I), and basally by a region that contained only E-cadherin labeling (arrows in Fig. 5E, F, G, H, I). These observations suggested that in these cells PLEKHA7 accumulates at the AJ belt, and extends slighly more apically than other AJ plaque proteins, suggesting that PLEKHA7 may partially overlap with TJ. However, although the linear distribution of PLEKHA7 and ZO-1 along the apical borders of bronchial cells was very similar and partially overlapping, close inspection of appropriately tilted sections showed that the two proteins were localized in adjacent, but separate areas of the junction (Fig. 5J-K), confirming that the localizations of PLEKHA7 and ZO-1 are distinct.

**Figure 6:**
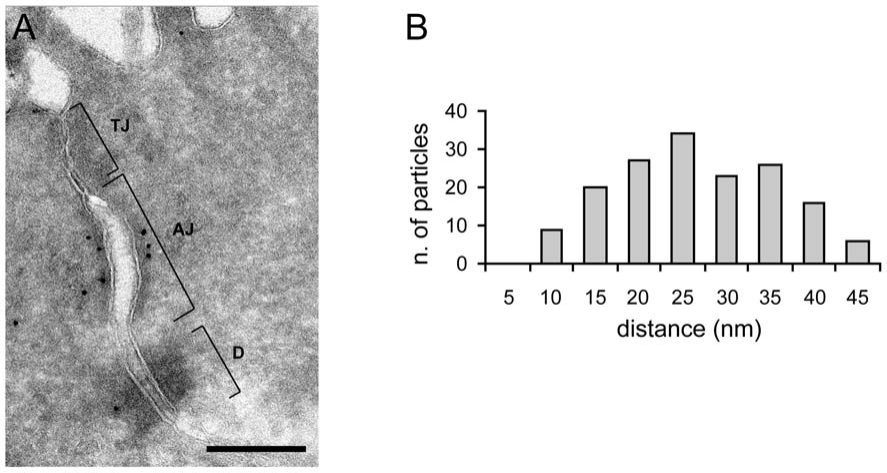
Ultrastructural localization of PLEKHA7 at AJ. (A) Immuno-gold electron microscopy localization of PLEKHA7 in mouse colon epithelial cells. Tight junction (TJ), adherens junction (AJ) and desmosome (D) are indicated with brackets. Note the accumulation of particles at the AJ. Bar = 300 nm. (B) Histogram showing the distribution of immuno-gold particles associated with the AJ, as a function of the distance from the plasma membrane (nm).

Finally, we determined the localization of PLEKHA7 at the ultrastructural level. Immunoelectron microscopy showed that PLEKHA7 labeling was accumulated at the cytoplasmic face of the AJ in intestinal epithelial cells (Fig. 6A). Among particles that were detected in the proximity of the plasma membrane (n = 241 out of 298 total), 78% were associated with the AJ, 3.7% with the TJ, 2% with the desmosome, 7.5% with the apical membrane and microvilli, and 7.9% with other areas. The mean distance of the gold particles from the AJ membrane was 28 nm (±12 nm, n = 188) (Fig. 6B), suggesting a close association of PLEKHA7 with the junctional membrane. Taken together, the immunofluorescence and immunoelectron microscopy data show that PLEKHA7 is localized at the AJ belt of epithelial cells.

## Discussion

Cell-cell junctions are heterogeneous in their morphology and molecular makeup, even within a single tissue [26], [27], [28], and mouse knockout studies show complex, redundant and non-redundant roles for junctional proteins [29], [30]. Thus, to clarify the role of junctional molecules in physiology and pathology, it is necessary to analyze systematically their expression, tissue distribution and localization. Here, we aimed to characterize the expression profile and the subcellular localization of PLEKHA7, a recently identified p120ctn-associated protein, that plays a fundamental role in tethering microtubule minus ends to the AJ [19].

Previously, the AJ localization of PLEKHA7 was inferred in cultured intestinal epithelial cells from the profile of distribution of PLEKHA7 immunofluorescent labeling along the lateral membrane, which was partially co-localized with E-cadherin [19]. However, nothing was known about the localization and distribution of PLEKHA7 in tissues. Here, we establish by immunoelectron microscopy that PLEKHA7 is localized at the AJ belt in epithelial cells. In addition, we show by immunofluorescence microscopy that PLEKHA7 is localized at cell-cell junctions in several epithelial tissues, with a subcellular localization and a tissue distribution that are distinct both from the TJ marker ZO-1, and also from several protein markers of the AJ. Unlike E-cadherin, p120ctn, α-catenin, and β-catenin, PLEKHA7 is absent from the “puncta adherentia” along lateral walls of epithelial cells, where a mobile pool of E-cadherin associates with many AJ plaque proteins [31], [32]. Why PLEKHA7 is not recruited to “puncta adherentia” by its interacting partner p120ctn is unclear, but one possibility is that the localization of PLEKHA7 and/or its interaction with p120ctn requires a simultaneous interaction with the microtubule cytoskeleton and/or associated proteins, which can only occur at the AJ belt. Conversely, the subcellular localization of PLEKHA7 in epithelial cells most closely resembles that of afadin, an AJ plaque protein that links the transmembrane protein nectin to the actin cytoskeleton, and also interacts with α-catenin [12], [25]. However, PLEKHA7 differs from afadin and ZO-1, because it is not clearly detectable in junctions of endothelial cells, that contain VE-cadherin, afadin and p120ctn [33]. Future studies should investigate in more detail the localization of PLEKHA7 in junctions of cells lining blood and lymph vessels, and in the “complexus adherens”, a unique type of adherens junction present in the lymph node, that contains VE-cadherin, as well as proteins of epithelial AJ, TJ and desmosomes [34]. Apart from the localization of PLEKHA7 in the outer limiting membrane of the retina, which contains heterotypic junctions between glial and neuronal cells, our observations suggest that the distribution of PLEKHA7 is essentially confined to epithelial junctions. The lack of PLEKHA7 labeling in heart intercalated disks, that contain cadherin and afadin [12] was unexpected, considering that PLEKHA7 mRNA and protein were detected in heart tissue by northern and western blotting. One possibility to explain this observation is that the epitopes recognized by our antibodies are modified in heart tissue, in a way that negatively affects immunofluorescent labeling. In summary, our observations indicate that PLEKHA7 is an AJ protein with a unique combination of subcellular localization and tissue distribution, since it is absent from puncta adherentia along the lateral walls of epithelial cells, and its pattern of tissue distribution is different from that of afadin.

We provide evidence that PLEKHA7 may exist in multiple isoforms within tissues. By northern blotting analysis we detect two major mRNAs species of PLEKHA7, with sizes of of ∼5.5 kb and ∼6.5 kb. In lysates of epithelial tissues, we identify by immunoblotting major polypeptides of M_r_ ∼135–145 kDa. A cross-reacting slower mobility polypeptide (M_r_ ∼240 kDa), prominently detected in the pancreas, is probably an unrelated protein recognized by anti-PLEKHA7 antibodies, based on two arguments. First, the M_r_ ∼240 kDa could not be immunoprecipitated from cultured epithelial cells. Second, bioinformatic analysis predicts two isoforms for human PLEKHA7 (both of M_r_ ∼127 kDa) and five isoforms for mouse PLEKHA7 (M_r_ ∼127, ∼144, ∼115, ∼117, ∼107 kDa), none of which corresponds to a ∼240 kDa molecular size. However, we cannot exclude that the slower migration of the M_r_ ∼240 kDa polypeptide may be due to post-translational modifications of the M_r_ ∼145 kDa polypeptide, that could occur in selected tissues. The functional significance of the PLEKHA7 isoforms, and the role of PLEKHA7 in cell differentiation should be clarified by future studies. For example, the observation that PLEKHA7 labeling in intestinal cells is stronger in the crypts rather than at the tip of the villi, suggests a modulated gradient of expression that correlates with cell differentiation, similar to that described for several AJ and TJ proteins [35].

PLEKHA7 has been proposed to regulate AJ stability through its ability to link the microtubule cytoskeleton to E-cadherin [19], and the E-cadherin/catenin/cytoskeleton complex is a crucial regulator of cancer invasion [36]. Thus, a question that should be addressed using cellular and animal model systems is whether AJ stabilization through PLEKHA7-dependent microtubule anchoring is important in cancer development and progression. Specific antibodies against PLEKHA7 will be a valuable tool for this purpose, and could also be useful in the histological typing and diagnosis of tumors.

## Materials and Methods

### Antibodies

To generate anti-PLEKHA7 antibodies, rabbits (New Zealand White, Pocono Farms Research Laboratory, www.prfal.com) and mice (Balb-c) were immunized by subcutaneous injection with an affinity purified, bacterially expressed recombinant fusion protein, comprising GST fused to a C-terminal fragment of human PLEKHA7 (residues 821–1121). To generate the PLEKHA7 construct in the expression vector pGEX4T1, we used polymerase chain reaction (PCR) amplification on a template of a full-length human PLEKHA7 cDNA in the pSPORT6 vector (IRAKp961M14168Q, RZPD www.imagenes-bio.de, corresponding to Genbank sequences ID BC033239.1 and GI:23138763). Truncated constructs of the antigen for epitope mapping purposes were also generated by PCR. The rabbit polyclonal anti-PLEKHA7 antiserum 20943 was used at a dilution of 1∶250 for immunofluorescence, and 1∶10,000 for immunoblotting.

Experiments and protocols on mice were authorized by the Geneva State Health Commission (Project name: “Production of mouse monoclonal antibodies by immunization with cell-cell junction proteins”, authorization number 1010/3356/1 by Dr. Astrid Rod of Direction Générale de la Santé, State of Geneva, 18.12.2007) and by the Ethics Committee for Animal Experimentation of the University of Geneva (Protocol 09-27). Monoclonal antibodies were obtained by fusion of NSO myeloma cells with lymphocytes isolated from axillar and inguinal lymphnodes of immune mice [37]. Hybridomas were screened by enzyme-linked immunosorbent assay (ELISA), immunoblotting and immunofluorescence microscopy, and two positive clones (16G2 and 378F1, that gave identical reactivity by immunoblotting) were isolated by limiting dilution, and were used as undiluted culture supernatants for immunofluorescence, and diluted 1∶100 for immunoblotting.

Other antibodies were: rat anti-ZO-1 (R40-76, from Dr. D. Goodenough, Harvard Medical School, 1∶50) [21], mouse anti-E-cadherin (Becton Dickinson Switzerland BD610181), rabbit anti-α-catenin (Sigma Switzerland C-2081, 1∶100), mouse anti-α-catenin (BD 610193, 1∶50), rabbit anti-β-catenin (Sigma C2206, 1∶100), mouse anti-p120ctn (15D2, from Dr. A. Reynolds, Vanderbilt University, USA, 1∶500) [38], rabbit anti-afadin (Sigma A-0224, 1∶500), rat anti-PECAM-1 (MEC13.3, BD 553370, 1∶50). TRITC-phalloidin was from Sigma (P-1951, 1∶1000). Fluorescein isothiocyanate (FITC)- and tetramethylrhodamine isothiocyanate (TRITC)-labeled secondary antibodies for immunofluorescence microscopy were from Jackson Immunoresearch Laboratories Europe (www.JIREurope.com).

### Northern blotting

A DNA probe of 1300 bp was generated using the DIG-PCR amplification kit (Roche, Switzerland) with the primers 5′-GAA GAG CCG AAT CCA CAT ATG-3′ and 5′-CTG GCG CTG CTG CCA CTC-3′ and the full length human PLEKHA7 cDNA as template. A human multiple tissue membrane (Clontech Europe, cat. 636818) was hybridized with 250 ng/ml probe in ExpressHybTM hybridization solution (Clontech) at 65°C overnight. Washes were performed twice with SSC 2× buffer (0.3 M NaCl, 30 mM sodium citrate, pH 7.0, containing 0.1% SDS) for 5 min at room temperature, and with SSC 0.5× buffer containing 0.1% SDS for 10 min at 60°C. Detection of hybridized probe was achieved with anti-DIG-AP antibody (Roche) followed by incubation in CSPD substrate (Roche), and exposure to X-ray film. The membrane was stripped with DEPC water containing 0.1% SDS at 100°C, and probed with human beta-actin probe (provided by Clontech), as a loading control.

### Cell culture and transfection

MDCKII cells were cultured in DMEM medium supplemented with 10% FBS, 100 U/ml penicillin, 100 µg/ml streptomycin and 1× minimal essential medium (MEM) non essential amino acids. mpkCCDc_14_ cells (mouse kidney distal tubule epithelial cells, a gift from Dr. E. Feraille, University of Geneva) were cultured in 1∶1 DMEM/HAMF12 medium, supplemented with 5 µg/ml insulin, 50 nM dexamethasone, 60 nM selenium, 5 µg/ml transferrin, 1 nM triiodothyronine, 10 ng/ml epidermal growth factor (EGF), 20 mM HEPES, 2 mM glutamine, 10% fetal bovine serum (FBS) and 20 mM D-glucose.

For transfection, Lipofectamine 2000 (Invitrogen, Carlsbad, California) was used, following the manufacturer's instructions. Myc-tagged full-length human PLEKHA7 was generated by cloning the full-length sequence (see above) in the vector pcDNA3.1myc-His. Cells were analyzed by immunofluorescence microscopy 48 h after transfection.

The generation and characterization of cells depleted of PLEKHA7 by expression of shRNA will be described elsewhere (Pulimeno et al, unpublished data).

### Immunoflurescence microscopy and immunoblotting

Cells on glass coverslips were fixed with cold methanol for 10 min at −20°C, washed three times with PBS, incubated with primary antibody (1 hr at 30°C), washed, incubated with secondary antibody (30 min at 37°C), washed and mounted with Vectashield medium (Reactolab, Servion, Switzerland). For immunohistochemistry, frozen sections (5 µm) were obtained from tissues included in OCT medium and snap-frozen in liquid nitrogen-cooled isopentane. Sections were air-dried, fixed with acetone at −20°C for 20 min, rehydrated in PBS, incubated with primary and secondary antibody (1 hr at 30°C, each followed by three washes in PBS), and mounted in Pro-Long anti-fade medium (Invitrogen) containing 1.5 µg/ml DAPI. Specimens were analyzed either with a Zeiss Axiovert S100 conventional epifluorescence microscope, or with a Zeiss 510 META confocal microscope. For confocal microscopy specimens were imaged using multi-track setting, in order to block bleed-through between channels.

For immunoblotting, cultured cell lysates were prepared as described previously [39]. To prepare lysates for immunoblotting, mouse tissues fragments (0.2–0.5 g) were homogenized in 1–1.5ml lysis buffer. For colon and kidney samples, we used Lysis Buffer A: 50 mM Tris-HCl, pH 7.4, 100 mM sodium pyrophosphate, 100 mM NaF, 10 mM EDTA, 10 mM sodium orthovanadate, 2 mM PMSF, 1% Triton X-100, 1% SDS, 10 mM DTT, protease inhibitor cocktail mix (1X, Roche). For other tissues, we used Lysis Buffer B: 50 mM Tris-HCl, pH 9.5, 2% SDS, 65 mM DTT, 10% glycerol. Tissues were homogenized at 4°C, and centrifuged at 4°C (A: 12,000 rpm 30 min, B: 45,000 rpm 2 hr). The supernatants were mixed 1∶1 with 2×SDS sample buffer, boiled and analyzed by SDS-PAGE. Gels were transferred onto nitrocellulose, and nitrocellulose membranes were blocked in TBS-milk (150 mM NaCl, 50 mM Tris-HCl pH 7.5, 0.1% Tween-20, 5% skimmed milk) for 1 hr, incubated with primary antibodies (16 hr 4°C), followed by HRP-coupled secondary antibodies (1 hr room temperature) and development with West Pico chemiluminescent substrate (Pierce).

### Immunoelectron microscopy

Segments of mouse intestine were fixed with 4% formaldehyde in 0.1 M phosphate buffer 20 min at room temperature, followed by overnight incubation at 4°C. Fixed tissue was cut in small cubic blocks (0.5 mm length), blocked in glycine (20 mM, in 0.1 M phosphate buffer) and infiltrated with 2.3 M sucrose in 0.1 M phosphate buffer. Ultrathin cryosections were prepared according to [40]. Briefly, sections (60–100 nm) were cut on a Leica Ultracut microtome at −120°C, picked up with 2.3 M sucrose and placed onto Formvar/carbon-coated copper grids. After blocking with 2% gelatine and then 1% BSA in PBS, sections were labelled with affinity-purified rabbit-anti-PLEKHA7 antibody (diluted 1∶30 in PBS with 1% BSA). Affinity purification of polyclonal antibodies was carried out by incubation of the serum with the antigen immobilized on nitrocellulose, followed by elution of antibodies with 200 mM Glycine-HCl, pH 2.8, and neutralization with Tris-HCl pH 7.5. Visualization of antibodies on sections was performed with 10-nm protein A-gold (obtained from G. Posthuma, Cell Microscopy Center, Utrecht, The Netherlands) diluted 1∶40 in the same buffer. Sections were post-fixed, stained and dried according to [41]. Specimens were examined on a Tecnai G2 electron microscope (FEI, Eindhoven, the Netherlands) at an accelerating voltage of 160 kV. Images were analyzed with ImageJ software.
